# Translation and validation of the shoulder instability-return to sport after injury (SIRSI) score in French

**DOI:** 10.1186/s40634-022-00470-2

**Published:** 2022-05-06

**Authors:** A. Louati, P. A. Bouche, T. Bauer, A. Hardy

**Affiliations:** 1 South Francilien Hospital, 40 avenue Serge Dassault, 91100 Corbeil-Essonnes, France; 2 Georges Pompidou European Hospital, 20 Rue Leblanc, 75015 Paris, France; 3 Lariboisière Hospital AP-HP, 2 rue Ambroise Paré, 75010 Paris, France; 4 Ambroise-Paré Hospital AP-HP, 9 Avenue Charles De Gaulle, 92100 Boulogne-Billancourt, France; 5 Sport Clinic, 36, Boulevard Saint-Marcel, 75005 Paris, France

## Introduction

Shoulder instability is a common event that is twice more frequent in young athletes than in the general population [40]. The protocol for surgical treatment is well defined and generally results in a good functional outcome with a low rate of recurrence [29]. Nevertheless, the rate of return to sport at the preinjury level of activity varies greatly (from 48% - 95.7% [10, 15, 24]) in an increasingly athletic population that expects more and more from treatment. After an episode of shoulder instability, a rapid return to sport at the preinjury level of activity is the priority for athletes. However, despite the good functional outcome after surgery, numerous athletes do not return to this level [13, 32].

This return to sport does not depend only on the functional status of the shoulder, but also on the patient’s psychological state and his/her readiness to return to sport [33]. The athlete’s recovery involves several steps described by Clement et al. [13]. After an injury, negative emotions associated with the severity of the injury and stopping sports are the primary feelings, followed by frustration during physical rehabilitation and finally at the return to sport, the patient feels doubt about his/her ability to play, anxiety, and fear of a new injury. During these different stages, it may be necessary to associate psychological support with the athlete’s physical training to return to the preinjury sport at the same level of activity [36].

This psychological preparedness cannot be measured using the usual scores, and questionnaires have been developed to quantify these factors and measure the psychological readiness of the patient to return to sport [6].

Webster et al. [43] developed the Anterior Cruciate Ligament-Return to Sport after Injury (ACL-RSI) score to quantify the psychological readiness of athletes to return to sport following ACL reconstruction. This questionnaire was translated and validated into French [11], then other versions of the questionnaires were adapted, such as the Shoulder Instability-Return to Sport after Injury (SIRSI) for shoulder instability [23] and the Ankle Ligament Reconstruction-Return to Sport after Injury (ALR-RSI) for the ankle [35].

The main goal of this study was to evaluate and confirm the reproducibility and validity of the French version of the SIRSI scale previously translated and validated by Gerometta [23], to measure the psychological readiness in athletes to return to their preinjury sport after surgical treatment of shoulder instability. The secondary goal was to look for a cutoff value of the SIRSI scale to discriminate patient able to return to sport.

The main hypothesis was that this tool would be reproducible and valid to evaluate these psychological factors before the return to sport, which would help practitioners make this decision and prevent the premature return to sport in patients who are not psychologically prepared.

## Materials and methods

### Validity and reliability of the final SIRSI-Fr

**Fig. 1:**
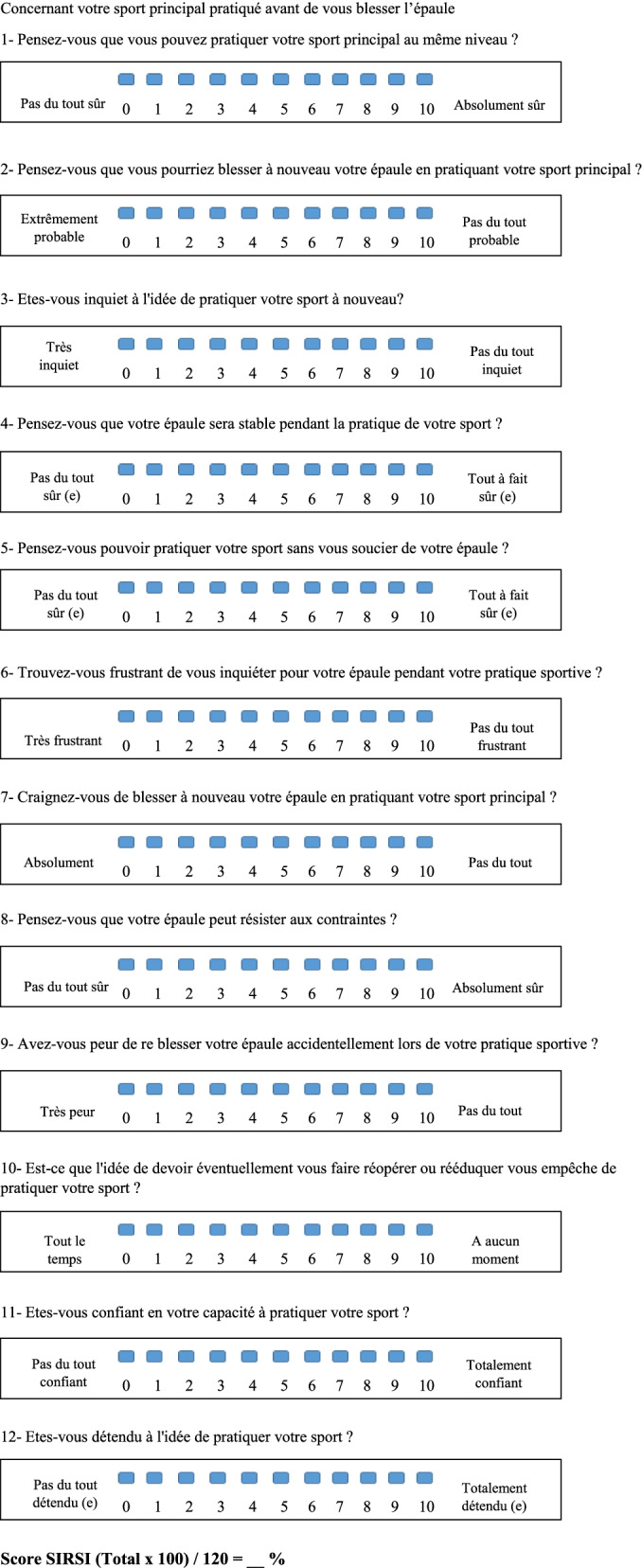
SIRSI-Fr Scale (French Version). Concernant votre sport principal pratiqué avant de vous blesser l’épaule. 1- Pensez-vous que vous pouvez pratiquer votre sport principal au même niveau?. 2- Pensez-vous que vous pourriez blesser à nouveau votre épaule en pratiquant votre sport principal?. 3- Etes-vous inquiet à l’idée de pratiquer votre sport à nouveau?. 4- Pensez-vous que votre épaule sera stable pendant la pratique de votre sport?. 5- Pensez-vous pouvoir pratiquer votre sport sans vous soucier de votre épaule?. 6- Trouvez-vous frustrant de vous inquiéter pour votre épaule pendant votre pratique sportive?. 7- Craignez-vous de blesser à nouveau votre épaule en pratiquant votre sport principal?. 8- Pensez-vous que votre épaule peut résister aux contraintes?. 9- Avez-vous peur de re blesser votre épaule accidentellement lors de votre pratique sportive?. 10- Est-ce que l’idée de devoir éventuellement vous faire réopérer ou rééduquer vous empêche de pratiquer votre sport?. 11- Etes-vous confiant en votre capacité à pratiquer votre sport?. 12- Etes-vous détendu à l’idée de pratiquer votre sport?. Score SIRSI (Total × 100) / 120 = __ %

Based on the French SIRSI-Fr **(**Fig. 1**)**, drafted by Gerometta [23], and validated according to the international COSMIN guidelines (Norms based on the Consensus Standard for the selection of instruments for the measurement of health status) [30], a prospective study was performed including patients who underwent surgery for shoulder instability from November 2018 to October 2020.

This study included two groups: a series of athletic patients who practiced different sports at different levels, aged 18 years or over who underwent surgery for shoulder instability, and a control group including athletes 18 years or older with no history of shoulder instability.

Exclusion criteria were: patient refusal, an incomplete response or a follow-up of less than 6 months.

The reference scales used were the Simple Shoulder Test (SST) [7, 25], West Ontario Shoulder Instability score (WOSI) [22, 27] with the different sections « Physical symptoms » /1000, «Sports/Leisure/Work » /400, « Lifestyle» /400 and « Emotions » /300, and the Walch-Duplay score [27, 39].

After a minimum follow-up of 6 months, each patient received an email with a link to the online questionnaire that was created and administered with Websurvey©. If the person did not respond a reminder was sent by email or, if necessary the patient was contacted by telephone. The SIRSI was filled out by all the patients twice, 5 days apart. The control group only filled out the questionnaire once. The questionnaires were only validated if all questions were answered.

### SIRSI

The development of the original version of the ACL-RSI was based on three elements that were correlated in relation to the return to sport in the literature: emotions, confidence in one’s performance and the evaluation of risk [41, 43].

Based on the ACL-RSI scale, SIRSI included 12 questions with 11 points on the Likert scale which had to be checked from 0 to 10 [11]. The total score had to be obtained by adding the values of the 12 responses then determining their relationship to 100 (× 100/12) to obtain a percentage.

High scores corresponded to a positive psychological response this study was approved by the CPP IDF VI Ethics committee. Informed consent was obtained from each patient.

### Statistical analysis

Continuous variables were presented as means or standard deviations. Binary variables were presented as the number of events and their percentages. The correlation (construction validity) between the SIRSI, the Walch-Duplay, the SST and the WOSI scores was estimated by the Spearman’s correlation coefficient. The correlation was considered “strong” (*r* > 0.5), «moderate» (0.5 < *r* < 0.3) or «weak» (0.3 < *r* < 0.1) [14].

The discriminant validity was tested by comparing the mean SIRSI score between « operated patients » and the « control » group by the Student t test, and between the group of patients who were operated and returned to their preinjury level of play and those who did not by the Mann–Whitney test.

Internal consistency was estimated by Cronbach’s alpha and the correlations between the different items on the SIRSI score were considered to be «excellent» if α > 0.90 [16, 17].

The reliability/reproducibility was evaluated by a test-retest with the Pearson correlation coefficient, the Bland and Altman diagram [9] and the intraclass correlation coefficient as *ρ* (CCIC); the reproducibility was considered to be « excellent » (*ρ* > 0.75), «good» (0.75 < *ρ* < 0.40) or «weak» (*ρ* < 0.40) [21].

The feasibility was estimated by the floor and ceiling effects corresponding to the percentage of patients with a minimum (0/10) or maximum score (10/10), respectively, for each question.

According to Terwee et al. [37]**,** the presence of a floor or ceiling effect of more than 15% indicates a problem in the validity of the contents when the elements of the questionnaire were being generated. A *p* value < 0.05 was considered to be significant.

The relationship between SIRSI sensitivity and specificity was calculated by an ROC curve for all possible cut-off values.

All of the analyses were performed with R software version 3.5.0.

## Results

### Description of the study subjects

The study included 55 « control » patients all athletes who played different sports at different levels, mean age 30.8 ± 10.2 years old, (22 men and 33 women) and 48 patients operated for shoulder instability corresponding to the inclusion criteria and who responded to all the questions in both questionnaires.

The sex ratio of the patient group was 2.42 (men: 70.8% - women: 29.2%). The mean age of patients at the first episode of shoulder instability was 23.1 ± 3.9 years old and 26.8 ± 7.5 years old when they were evaluated, or a mean 3.8 ± 3.6 years after the first episode of shoulder instability **(**Table 1**)**.

**Table 1:** Participants vs control group

Parameters	Values	N	Statistics*	N	Statistics*	*P*. Value
		48	Intervention	55	Control	
Age (years)		48	26.81 (7.53)	55	30.86 (10.19)	0.06
Gender	Women	14	29.2%	33	60%	< 0.01
	Men	34	70.8%	22	40%	
BMI (Kg/m^2^)		48	22.74 (2.81)	55	22.65 (3.262)	0.83
Dominant arm	Right	41	85.4%	52	94.5%	0.18
	Left	7	14.6%	3	5.5%	
Operated side	Right	29	60.4%			
	Left	19	39.6%			
Hyperlax	No	30	62.5%			
	Yes	18	37.5%			
Sports Level	Competition	16	33.3%	4	7.3%	< 0.01
	Leisure	28	58.3%	32	58.2%	
	No sport	4	8.3%	19	34.5%	
Sport’s type	Overhead	12	25.0%	6	10.9%	< 0.01
	Others	15	31.2%	22	40%	
	Contact	19	39.6%	8	14.5%	
	No sport	2	4.2%	19	34.5%	
Total SIRSI score		48	68.96 (17.91)	55	85.16 (8.16)	< 0.01
Total Walch-Duplay score		55	64.48 (18.97)	55	81.84 (12.19)	< 0.01
SST score		55	9.27 (2.19)	55	9.509 (1.386)	0.94
Total WOSI score		55	797.9 (306.5)	55	364 (169.4)	< 0.01
Were you able to resume sport?	No	14	29.2%			
	Yes	34	70.8%			

Shoulder instability occurred while playing sports in most patients 45/49 (91.8%). Patients played a competitive sport in 33.3% of the cases and a leisure sport in 58.3%. None were professional athletes. The injury occurred while playing a contact sport in 39.6% of the cases and a combat sport in 25%.

The dominant shoulder was involved in 54% of the cases. Eighteen (37.5%) of the patients presented with constitutional hyper laxity. The episode of instability included dislocation in 37 (75.5%) and subluxation in 12 (24.5%).

All patients were surgically treated for their injury with the Latarjet procedure, which was performed after the first episode of instability in 19 (38.7%) patients.

The mean patient follow-up was 18.04 ± 7.9 months.

### Return to sport

Thirty-four (70.8%) patients returned to sport after a mean 4.4 ± 1.2 months of follow-up. Fifteen of these patients (44.1%) returned to the same level of activity as before injury at 4.3 ± 0.3 months, four (11.7%) at a higher level, eleven (32.3%) at a lower level of the preinjury sport and four (11.7%) changed sport. Fourteen (29.2%) patients had not returned to sport at the final follow-up.

### Construct validity

SIRSI was found to be significantly correlated to all the reference scores. This correlation was moderate for the Walch-Duplay (*r* = 0.39) and moderate but stronger for the total WOSI and its elements (*r* = 0.48). The correlation with the SST was weak (*r* = 0.19) **(**Table 2**)**.

**Table 2:** correlation SIRSI and other scores

	SIRSI (/120)	Total Walch-Duplay (/100)	SST score (/12)	Total WOSI (/2100)
Coefficient	68.96 (17.91)	64.48 (18.97)	9.27 (2.19)	797.9 (306.5)
Spearman		0.39 [0.12–0.60]	0.19 [−0.12–0.47]	0.48 [0.23–0.68]

The SIRSI is divergent correlated with BMI (spearman coefficient = − 0.25 [− 0.48;0.01]) and with VAS score (spearman coefficient = − 0.29 [− 0.57;0.04]).

### Discriminant value

A significant difference was observed between the « patient » group and the « control » group and between the subgroup that returned to sport at the same level of activity and the rest of the patients **(**Table 3**)**.

**Table 3:** Mean score of the SIRSI group who returned to sport vs no return

« same level of play» group	« lower level or stopped » group
78.69 (11.69)	65.34 (18.59)
*P.value = 0.03*	

### Internal consistency

The internal consistency of the scale which measures the consistency among the 12 items within the questionnaire was «good» with a Cronbach’s alpha of 0.83 [16, 17].

### Reliability

**Fig. 2:**
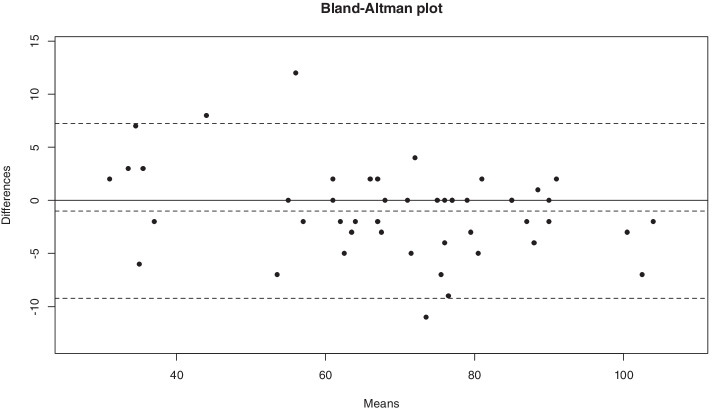
Reproducibility of the SI-RSI by Test-Retest Bland-Altman Diagram

The mean SIRSI score for the first test was 57.46% (±14.9) and 58.3% (±16) for the second test. The reproducibility was «excellent» with an intraclass correlation coefficient of *ρ* = 0.97 [1.96–0.99], *p* < 0.00001 **(**Table 4**,** Fig. 2**)**.

**Table 4:** : Reproducibility of SIRSI score by Test-Retest

Coefficient	SIRSI 1 (/120)		SIRSI 2 (/120)	
	68.96/120	57,46%	69.96/120	58,3%
	(17.91)	(14,6%)	(19.23)	(16%)
ICC			*ρ* = 0.97 [0.96–0.99]

### Feasibility

For the floor effect, the mean percentage of response at 0/10 for each question of the SIRSI score was 4.5% (±5.3).

For the ceiling effect, the mean percentage of response at 10/10 for each question of the SIRSI score was 10.2% (±9).

All of the patients responded to all the questions of the SIRSI score. There were no missing data.

### Cutt off

**Fig. 3:**
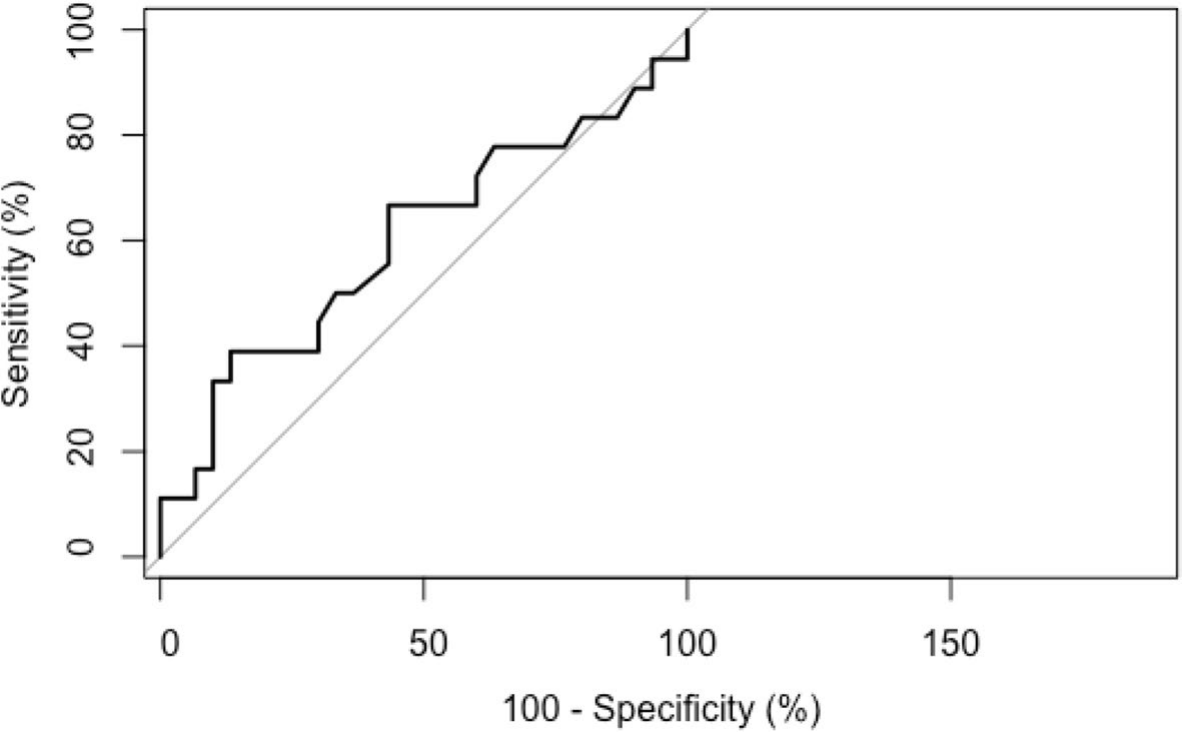
Relationship between SIRSI specificity and sensibility by ROC curve

The ROC curve shows a cut off at 60.5 for a sensitivity of 39% and a specificity of 86.6% to discriminate patient who were able to return to sport **(**Fig. 3**)**.

## Discussion

The main result of this study was that this French version of the SIRSI score is a valid, reliable and reproducible tool to identify unstable and non-unstable patients. It is effective and specific in discriminating between those who are psychologically ready to return to sport or not, following surgery for an episode of shoulder instability.

Warth et al. [41] reported that for 95.5% of the patients between 18 and 78 years’ old who undergo shoulder surgery, the most important demand is the return to sport. The return to sport at the same level of activity is therefore the main evaluation criteria to define successful surgical management of patients with shoulder instability [12, 24, 26], and it is essential for the surgeon to be able to identify the risk factors associated with not returning to sport. The systematic review of the literature by Watson et al. [42] identified consensus criteria for the return to sport following objective dislocation of the shoulder: a painless shoulder, muscular strength similar to the contralateral side and enough range of movement to play the patient’s preinjury sport. Despite successful surgery and physical therapy, treatment fails in certain patients or their performance is poorer for no objective physical reason [18]. Indeed, patients must not only be physically ready but also psychologically prepared to return to sport after surgery [35].

Numerous functional scores and several questionnaires have been developed to evaluate functional recovery of the upper limbs, in particular the shoulder [7, 10, 22, 28, 34, 39]. However, the clinical value of these tools is not sufficient to authorize patients to return to sport. Indeed, they do not always correspond to the patient’s actual recovery of performance because they provide information on objective functional status of the shoulder without taking into account the patient’s psychological state. Arden et al. **[**2, 3, 4] showed that the return to sport rate was better in patients with positive psychological responses before ACL reconstruction surgery. Similar results were reported by Tjong et al. [38] in patients operated for shoulder instability. These studies confirm the necessity of taking into account psychological factors and not only functional scores in the decision to return to sport. It is therefore essential to identify specific questions to better understand an athlete’s real recovery and analyze their athletic capacities and psychological readiness [5, 8].

Gerometta et al. **[**24] reported that a patient’s psychological readiness should be taken into account in the decision to return to sport [2]. The SIRSI score, like the ACL-RSI [11], and the ALR-RSI for ankle instability [35], makes it possible to identify patients who will have psychological difficulty returning to their preinjury sport. Indeed, a significant difference was found in patients’ psychological readiness, quantified by the SIRSI. This score was lower in athletes who did not return to sport at the preinjury level after an episode of instability.

Three questionnaires were used to validate the SIRSI: the Walch-Duplay, the WOSI and the SST scores. The SIRSI scale specifically evaluates the impact of psychological factors on the return to sport following surgery for shoulder instability. There was a significant positive correlation between the SIRSI and all the other questionnaires used. Although it is moderate, the correlation with the WOSI score was the strongest (*r* = 0.48), while the correlation with the Walch-Duplay was *r* = 0.39 and the weakest correlation was with the SST, *r* = 0.19. The weak correlation with the SST can be explained by the low specificity of the latter score, in particular for shoulder instability [7, 25, 31].

The present study found lower correlation values with WOSI and Walch-Duplay scores than the Gerometta et al. study [23]. All of the patients in Gerometta’s study were professional or competitive rugby players, while our patients played a variety of sports, 58% of our patients played a recreational sport and only a third at a competitive level. This could explain the difference in motivation to return to sport and the psychological state of the patient regarding the return to sport, thus explaining the weaker correlations than those found by Gerometta [23].

On the other hand, the WOSI was developed as a self-administered quality of life questionnaire specific to shoulder instability **[**34]. It allows a subjective self-assessment of the patient summarizing his opinion (satisfaction/disappointment). Critics suggest using the WOSI only for non-athletes and amateur athletes [20, 27, 31, 34], since the return to the initial sporting level, which would be the best measure of outcome in athletes, was not taken into account in the WOSI [27]. Hence the interest of a questionnaire specifically dedicated to the evaluation of psychological preparation for returning to sport.

Furthermore, the Walch-Duplays score, both objective and subjective, is the benchmark score in Europe for assessing shoulder instability [19]. The correlation between the Walch-Duplay score and the WOSI has been shown to be strong in terms of overall scores. The limit lies in the evaluation of the range of motion of the shoulders, which requires a physical examination [27]. This element is missing in self-administered questionnaires like the SIRSI. The subjective nature of this score naturally increases the scattering of the data and their lack of distinction [34].

The discriminant value of SIRSI was also confirmed. This questionnaire correctly distinguished patients who underwent surgery for shoulder instability from those without instability, and patients who returned to their preinjury sport at the same, or a higher level, from those who did not return to sport or who returned at a lower level. The floor and ceiling effects were acceptable (< 15%). This scale successfully evaluated the return to sport in the target population for which it was developed, confirming the hypothesis.

This study has several limitations. Although it is a prospective study the follow-up is short (a minimum of 6 months). As shown by Cicotti [12] and Gerometta [23, 24], this is not always enough to evaluate the return to sport at the preinjury level. It is also important to note that the rate of and mean time until the return to sport, as well as a return at the same level of activity might have been longer if the follow-up was longer.

Moreover, the SIRSI was developed as an adaptation of the ACL-RSI score. Nevertheless, the questions are not specific for a certain body joint, and can be easily applied to other joints involved in sports injuries [35].

To be definitively validated, the score must be validated by experts and by patients of different nationalities to be sure that its items are understood by all.

Although the choice of a quantitative questionnaire might seem simplistic compared to a psychological assessment, quantitative simplification of the responses provides a standardized, quantified and reproducible analysis of the patient’s psychological state in relation to the return to sport after an injury. The use of a questionnaire is then easier for doctors and surgeons in their daily practice. Indeed, allowing a patient to return to sport is a difficult decision, and there is no consensus on the subject [1]. Moreover, the practitioner must be able to recognize the factors that could prevent a return to sport and provide specific advice to overcome the athletes fear.

## Conclusions

The French version of the SIRSI is a valid, discriminant, coherent and reproducible score to evaluate the psychological preparation of the patient to return to the preinjury sport after an episode of surgically treated shoulder instability. It could be used more extensively and as a tool to authorize the athlete to return to sport. We recommend using the different scores (subjective and objective) to measure all of the functional disorders. Their complementary use makes it possible both to assess the patient’s quality of life, the objective assessment of the surgeon and the psychological preparation of the athlete for return to sport.

## Data Availability

available.
